# Immunohistochemical Characterization of Reactive Epithelial Changes in Odontogenic Keratocysts

**DOI:** 10.1007/s12253-019-00749-3

**Published:** 2019-10-18

**Authors:** Dorottya Cserni, Tamás Zombori, Anette Stájer, Annamária Rimovszki, Gábor Cserni, Zoltán Baráth

**Affiliations:** 1 Department of Prosthodontics, Faculty of Dentistry, University of Szeged, Tisza Lajos krt 64-66., Szeged, H-6720 Hungary; 2 Department of Pathology, University of Szeged, Állomás u. 1, Szeged, H-6725 Hungary; 3 Department of Pathology, Bács-Kiskun County Teaching Hospital, Nyíri út 38, Kecskemét, H-6000 Hungary

**Keywords:** Odontogenic keratocyst, Immunohistochemistry, bcl2, Cytokeratin 10, Cytokeratin 17, Cytokeratin 19

## Abstract

Odontogenic keratocysts (OKCs) have a diagnostic thin epithelial lining characterised by a linear epithelial connective tissue interface generally lacking inflammatory changes, basal palisading of the nuclei and a wavy parakeratotic layer on the surface. This typical epithelium may convert to a thicker non-keratinizing one with rete pegs and a relatively flat surface after operative decompression. The aim was to characterize this type of epithelial change by immunohistochemistry for bcl2, keratin17, 10 and 19. Eleven out of 33 archived OKCs demonstrated an altered epithelium related to previous biopsy, decompressing drainage or inflammation. The typical basal bcl2 staining was lost in 10/11 cases; transepithelial CK17 was lost or markedly reduced in 9/11 cases. CK10 displayed a segmental upper layer staining in OKCs, and its loss or partial loss in the altered epithelium did not differ from negative areas of OKCs. CK19 displayed various staining patterns in the altered epithelium from lost to maintained in a patchy transepithelial distribution, the latter of which did not differ from the typical OKC staining pattern. Three of four non-keratinizing epithelial linings with basal palisading displayed immunostaining reminiscent of typical OKC epithelium. The lack of a typical epithelium is not sufficient to exclude the diagnosis of OKC if the sampling is not generous (e.g. biopsy), and the presence of non-keratinizing epithelium with basal palisading and an immunophenotype characteristic of OKC (basal bcl2, patchy or diffuse CK17 and upper layer CK10 positivity) may be consistent with the OKC diagnosis even in the absence of typical epithelial lining.

## Introduction

Odontogenic keratocysts (OKCs) were first described by Philipsen in 1956 [1]. Not much later, in 1960, they were recognized as a common feature of the Gorlin-Goltz syndrome, the nevoid basal cell carcinoma syndrome, although the jaw cysts of the syndrome were not identified as “OKCs” in the original description [2]. They are currently classified as non-inflammatory odontogenic cysts in the WHO classification of head and neck tumours [3], but in the previous edition, they appeared under the name of keratocystic odontogenic tumour (KCOT), and this was principally related to their frequent mutation in the PTCH1 (patched) tumour suppressor gene, a key player in the sonic hedgehog (SHH) pathway [4, 5]. The re-reclassification was made because the evidence favouring the neoplastic nature of this tumour or tumour like condition was considered insufficient at the time. Indeed, other odontogenic cysts have been described to harbour PTCH1 mutations or express related proteins [6–8]. Independently of the neoplastic nature, PTCH1 mutations are common in OKCs [5], and owing to this background, vismodegib, an inhibitor of the SMO (smoothened) component of the SHH pathway has proven of efficacy in reducing the size of syndromic OKCs to nearly complete regression [9]. Whatever their nature, OKCs have a tendency to recur, and despite the favouring of conservative surgical approaches [10], owing to their destructive local growth, they may lead to radical surgery [11]. Therefore, the diagnosis of OKC needs caution to avoid both underdiagnosis and overdiagnosis.

**Fig. 1:**
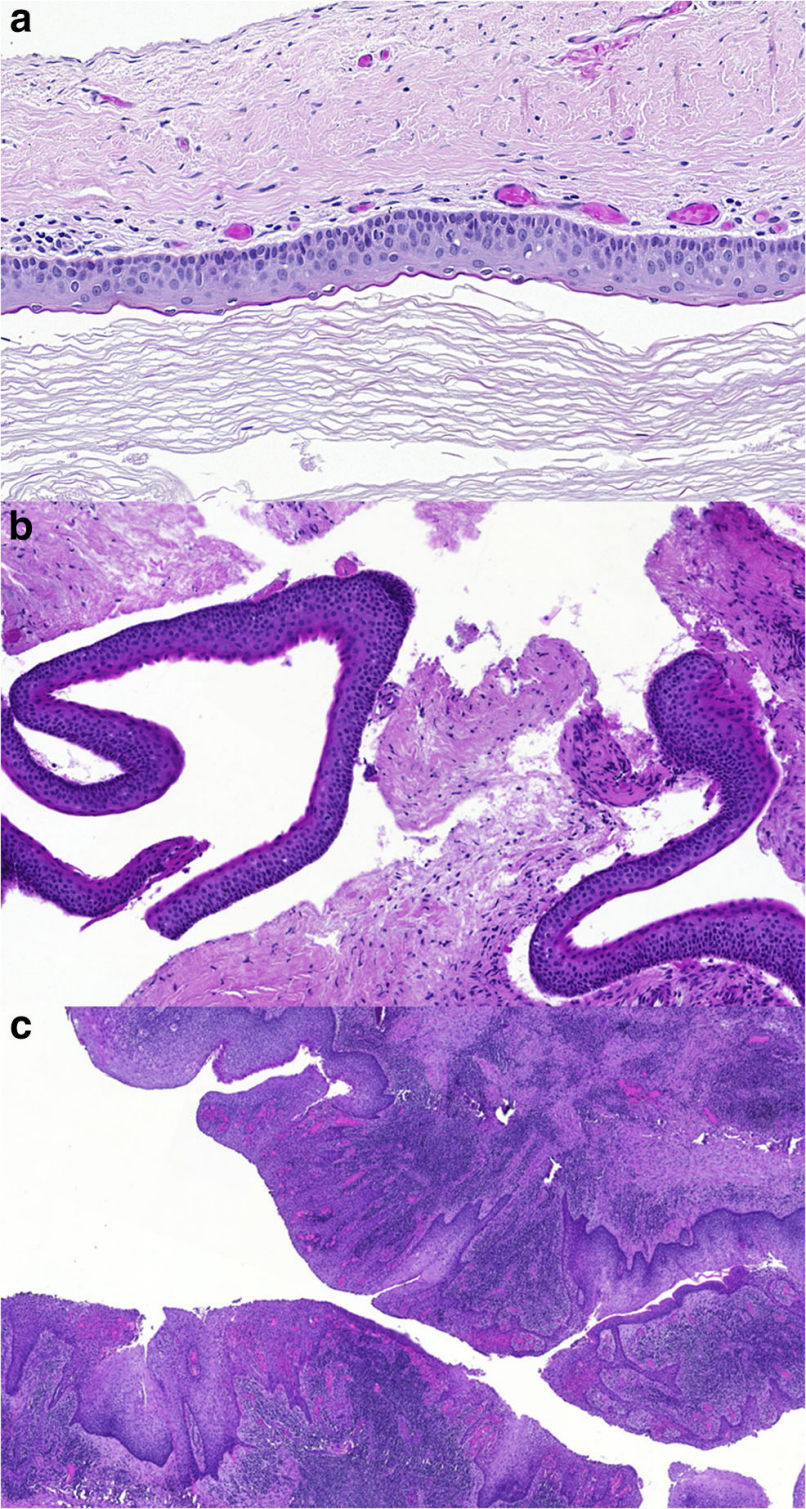
Typical and non-typical epithelium in OKCs. A: typical features including thin epithelium with linear epithelial connective tissue interface, basal palisading and upper wavy parakeratotic layer (note two parakeratotic nuclei in between the horny scales; Case K1); B: biopsy particle showing typical epithelium detached from the supporting connective tissue which is not an uncommon feature in biopsies (case K5); C: surgical specimen displaying large area of thicker epithelium with loss of keratinization, superficial waviness and partial loss of basal palisading and underlying inflammation (case K5). (Haematoxylin and eosin, HE; A: ×40, B and C: ×20)

OKCs can occur anywhere in the jaws, but have a predilection for the angle and ramus of the mandible. On radiology, they occur as radiolucent lesions often with a scalloped contour and peripheral sclerosis. They have a very characteristic histology: they have a few-cell-thick, i.e. thin, layer of squamous epithelium, with a relatively sharp epithelial – stromal interface, basal palisading of epithelial cell nuclei, wavy upper layer of parakeratotic epithelium. The epithelial – connective tissue interface is generally devoid of inflammation. When this typical histology is present (Fig. 1a), the histological diagnosis of OKC is straight forward, but sometimes the characteristic features are seen only focally, and the typical epithelial lining is lost, denuded or transformed due to inflammation and/or previous opening of the cyst. This may make the diagnosis of OKC difficult or impossible. Personal and consultation experience with this diagnostic difficulty (e.g. a typical OKC epithelium in the biopsy, and only focal presence of this epithelium with dominant altered variant in the surgical specimen 2 months later; Fig. 1b and c) has partly prompted the present work.

Immunohistochemistry (IHC) has been used to characterize and distinguish the epithelium of OKCs from other cysts of the jaws, including developmental and inflammatory cysts or cystic neoplasms and also the orthokeratinizing odontogenic cyst (OOC), which was segregated from the OKC group and appeared as a distinct and different entity only in the 4th edition of the WHO classification [3].

The typical epithelium of OKCs (including primary, recurrent and syndromic OKCs) has been reported to demonstrate bcl2 labelling of the basal layer unlike OOCs, dentigerous and radicular cysts, which all lack this staining [12]. This phenomenon may be related to the SHH pathway (often activated in OKCs due to PTCH1 mutations), as one of its downstream components, GLI2 acts directly on bcl2 transcription [13]. Another typical feature of OKCs is the CK17 positivity of their epithelium, principally in the intermediate (spinous) layer with lesser staining of the cells in the basal and/or sometimes the superficial layer; this staining seems to be completely missing from OOCs [14, 15] and dentigerous or radicular cysts [15]. Cystic ameloblastomas share the bcl2 staining with OKCs [12], and are also positive with CK17, but this staining has been reported to be limited to the superficial layers, in contrast with the transepithelial staining noted in OKCs [14]. CK19 shows a staining very similar to that of CK17 in OKCs, i.e. it demonstrates a transepithelial staining most of the time, but basal cells or sometimes suprabasal and spinous cells might not stain for this cytokeratin giving a more patchy labelling pattern. This feature may again be useful in the distinction from OOCs generally not labelled at all or showing CK19 staining limited to the basal layer(s) [15, 16]. However, dentigerous cysts and radicular cysts also usually demonstrate CK19 positivity in all layers of their epithelium [15], and therefore CK19 may not be an ideal marker for differentiating between odontogenic cysts. CK10 has also been studied in the differential diagnostic setting. It was found to be positive in the spinous layer and superficial layer of all OOCs, in the superficial layer of about 10% of 25 OKCs, while being completely negative in 15 dentigerous and 15 radicular cysts [15]. The cytokeratin profiling of OKCs has not only differential diagnostic implications, but has led to stating that they may originate from the dental lamina, with which they share staining patterns [14].

The aim of the present study was to analyse the histological features of OKCs, identify and characterize encountered epithelial changes occurring in them.

## Materials and Methods

Specimens sent to the Department of Pathology of the Albert Szent-György Medical Centre of the University of Szeged with “cyst of the jaw” coded diagnoses and “cyst” labelled specimens sent to the Department of Pathology of the Bács-Kiskun County Teaching Hospital by the Head and Neck Surgery Units of these institutions were collected and screened for the diagnosis. Cases with a diagnosis of OKC or KCOT were reassessed, and those displaying the characteristic features of OKC (at least focally in a specimen or one specimen from several consecutive specimens) were analysed further. Case histories of the patients with a confirmed OKC diagnosis were studied, and in case of recurrent OKCs, steps were made to recover the previous material, too.

During the histopathology review, care was taken to identify areas of non-typical epithelium in the cysts and the presence of inflammation, outpouchings and/or pericystic odontogenic epithelium islands that could predispose to recurrence.

IHC to analyse possible differences between the original and altered cyst lining epithelium was performed with the antibodies and details listed in Table 1. All reactions were carried out on 3–4-μm-thick sections from the archived formalin-fixed and paraffin embedded tissue blocks. For the IHC, heat induced epitope retrieval was used (45 min in 98 °C water bath at pH 6 with citrate buffer for bcl-2 and CK19; 6 min at high pressure at pH 9 with TRIS-EGTA buffer for CK10). The incubation with the primary antibodies lasted 60 min at room temperature, and the reactions were developed with Vector ImmPRESS (Vector Laboratories, Burlingame, CA, USA) for 30 min, also at room temperature. CK17 IHC was run on a Bond Max autostainer (Leica Biosystems, Wetzlar, Germany) with epitope retrieval with ER1 solution for 20 min and incubation time of 15 min.

**Table 1:** Antibodies and their sources used in the study

Antibody	Clone (Source)	Dilution
bcl2	3.1 (Novocastra/Leica, Newcastle, UK)	1:80
CK10	SP99 (Master Diagnostica, Granada, Spain)	RTU
CK17	E3 (Novocastra/Leica, Newcastle, UK)	RTU (1:2)
CK19	A53-B/A2.26 (Cell Marque, Rocklin, CA, USA)	1:500

*RTU* ready to use

The study was approved by the Ethical Committee/Human Investigation Review Board of the Albert Szent-Györgyi Medical Centre.

## Results

From January 2015 to June 2019, 256 “jaw cysts” and 143 “cysts removed by head and neck surgeons” had been seen at the two departments of Pathology. These were reviewed for their diagnosis and selected cases with their previous biopsies were further reviewed by reassessment of all available histology slides. Altogether 33 OKCs of 22 patients were identified. The median age of the patients at presentation was 35 years (range 10–70); if the 4 patients older than 60 are excluded, the median age becomes 29. The male to female ratio was 16/6. Two patients had Gorlin-Goltz syndrome; a female patient presented with a synchronous bilateral mandibular OKC, followed by a metachronous left maxillary OKC, whereas a male patient presented with synchronous bilateral mandibular (the left sided being double in the molar and the premolar region) and left maxillary OKC. Two male patients had synchronous dual unilateral OKCs, one of them suffered a recurrence 6 years after cystectomy. Two further male patients had one and two recurrent OKCs, respectively; the first lesion recurred one year after cystectomy, whereas the second had recurrences two and three years following the first cystectomy.

Seven lesions of 6 patients were identified in the maxilla, whereas the rest was in the mandible, many appearing in the angle extending to the body and/or the ramus. The clinical presentation could be recovered in only 12 patients: 5 were asymptomatic with the OKCs discovered on radiographs performed for other reasons, 5 patients noted minor swelling, whereas 2 had mild pain in the region of the cyst. One of the patients with swelling, also had paraesthesia related to OKC. Radiographs were available for review in 12 cases (11 patients): all lesions were radiolucent and eight had at least partially uneven contours. Overall, on the basis of the clinical and radiological presentation, OKC was suggested as diagnosis or as a differential diagnostic entity in 19 cases.

Six OKCs of five patients were first diagnosed with incisional biopsy, and were removed later on, either following temporary preoperative drainage or without such conservative attempt to treat. Three of these lesions, including one drained, showed epithelial change and the presence of epithelium not typical for OKC. Furthermore, 8 samples (including two of the biopsy specimens just mentioned) had foci of inflammation and epithelium not typical for OKCs at the site of the inflammatory reaction (Table 2).

**Table 2:** Differential staining of the typical and the non-typical OKC epithelium

IHC staining:			bcl2		CK17		CK10		CK19	
Case	Description	Interval	typical	non-typical	typical	non-typical	typical	non-typical	typical	non-typical
K1 ^a^	No I to I	na	b/sb	lower mid ^a^	trans	trans to lower mid ^a^	spf/usp to neg	spf/usp to neg ^a^	trans	trans
K1 ^c^	B to S	12.5	b/sb	neg ^c^	trans	neg ^c^	spf/usp to neg	neg to 1–1 usp to patchy usp/spf	trans	ba to neg
K4	No I to I	na	b/sb	neg	trans	neg	spf/usp to neg	neg	trans	trans
K5 ^b^	B to S	2	b/sb	neg to weak b/sb ^b^	trans	patchy trans to neg ^b^	spf/usp to neg	spf/usp to neg ^b^	trans	trans to neg
K6	No I to I	na	b/sb	neg	trans	patchy pos to neg	spf/usp to 1–1 usp to neg	neg to 1–1 usp	trans	trans
K7	No I to I	na	b/sb	neg	trans	neg	spf/usp to 1–1 usp to neg	neg	trans	trans
S2a	No I to I	na	b/sb	neg	trans	neg to weak patchy pos	spf/usp to neg	spf/usp to neg	trans to neg	neg
S8	No I to I	na	b/sb	neg	trans	neg to 1–1 to weak patchy	spf/usp to neg	neg to 1–1 sp. to patchy pos	trans	ba to 1–1 to neg
S13	No I to I	na	b/sb	neg	trans	trans	spf/usp to 1–1 usp to neg	neg	trans	trans to neg
S13	B to S	3	b/sb	neg	trans	neg	spf/usp to 1–1 usp to neg	neg to 1–1	trans	trans to 1–1
S15c ^b^	No I to I	na	b/sb	neg to b/sb ^b^	trans	b/sb to neg ^b^	spf/usp to 1–1 usp to neg	1–1 to neg ^b^	trans to neg	trans to neg

Description: B: biopsy, I: inflammatory changes, S: surgery/surgical specimen; mo: month, na: not applicable; Layers: b: basal, mid: middle, sb: suprabasal, sp.: spinous, spf: superficial, trans: transepithelial (different intensity but basal, suprabasal, upper spinous and superficial layers stained diffusely or partially with possible patches of unstained areas), usp: upper spinous; neg: negative, pos: positive, 1–1: few scattered cells;

Non-keratinizing squamous epithelium with basal palisading (probably representing transition from typical to non-typical lining epithelium) closely matching the typical OKC epithelium staining [(a): only palisading type present], (b) whereas non-keratinizing squamous epithelium without basal palisading deviating from it. (c): palisading non-keratinizing squamous epithelium present, but showing no overlap in staining with the typical OKC epithelial lining

On histology, owing to the selection criteria, all OKCs showed the characteristic features described in the introduction: linear stromal-epithelial interface, palisading of basal cells, thin epithelium with at least focally wavy surface plus parakeratosis. The altered epithelium, in contrast, showed complete or partial loss of basal palisading, often a wavy stromal epithelial interface with rete pegs, loss of keratinisation and loss of waviness on the surface (Fig. 1c). Denudation and inflammatory changes were also encountered in these specimens.

**Fig. 2:**
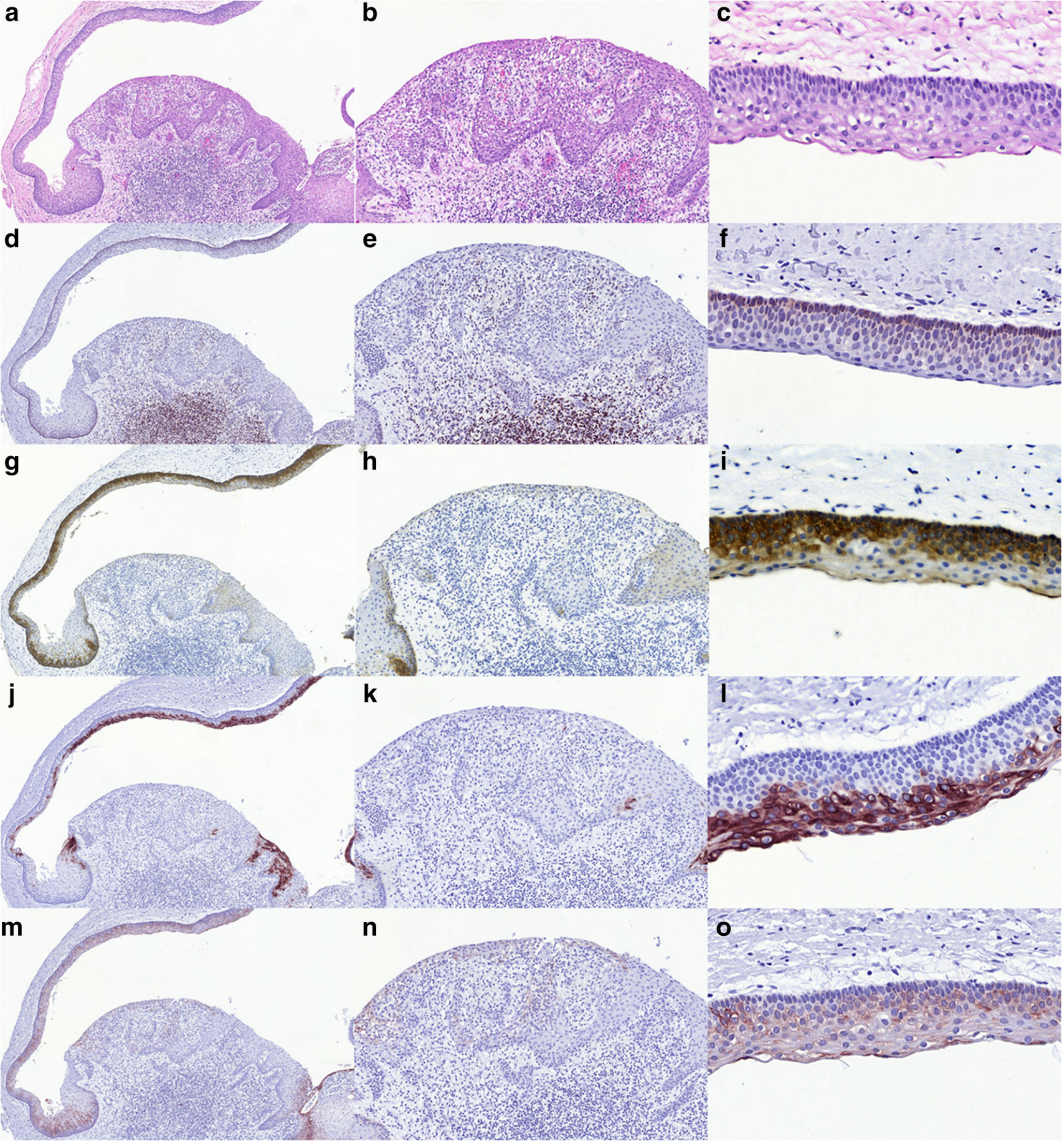
Inflammation associated changes in the epithelial lining of OKCs. An area in Case S8 demonstrating inflammation with overlying non-keratinizing epithelium. The first column (A, D, G, J, M, all ×10) gives an overview, the second (B, E, H, K, N, all ×20) highlights the altered epithelium, and the third (C, F, I, L, O, all ×60) represents the typical OKC lining epithelium from the top of the low magnification image in the first column. The pictures in each row represent different stainings: HE, bcl2, CK17, CK10, CK19 from row one to five). Note lost (or markedly reduced) bcl2, CK17, CK10 and CK19 staining in the middle column

**Fig. 3:**
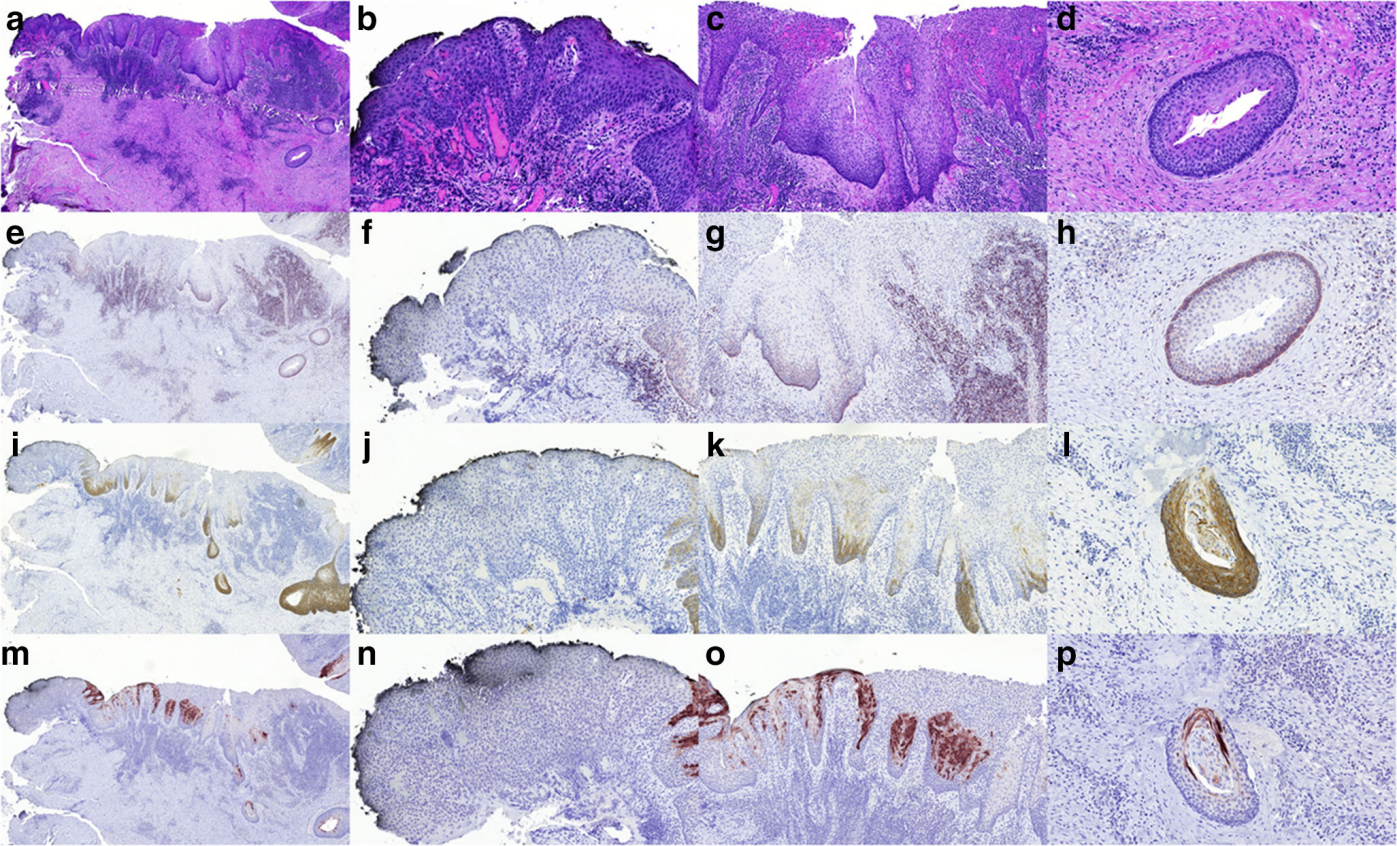
Transition from relatively thick non-keratinizing squamous epithelium with palisading to one without basal palisading. An area in Case K5 demonstrating inflammation with overlying non-keratinizing epithelium. The first column (A, E, I, M, all ×4) gives an overview of the altered epithelium with details in the subsequent columns. The second (B, F, J, N, all ×15) highlights the altered epithelium without palisading and loss of bcl2, CK17 and CK10 staining; the third (C, G, K, O, all ×10) represents the altered non-keratinizing epithelium with maintained basal palisading, bcl2, CK17 and CK10 staining; whereas the fourth (D, H, L, P, all ×20) shows one of the small daughter cysts or outpouchings of the OKC with the characteristic epithelium. The pictures in each row represent different stainings: HE, bcl2, CK17, CK10, from row one to four). Note bcl2 positivity in the inflammatory infiltrate

The results of the IHC are summarized in Table 2. The typical IHC phenotype of OKCs included diffuse basal (often with a weaker suprabasal) bcl2 positivity with no staining of the upper half, two thirds of the epithelium. CK17 positivity generally showed a diffuse or patchy transepithelial staining including focal basal and relatively diffuse suprabasal, spinous and again focal superficial layer labelling. CK10 showed a mosaic like staining pattern composed of variable areas of the superficial and upper spinous layers stained (often marked, but at areas only limited to a few cells) and completely negative areas. CK19 demonstrated a pattern similar to CK17, with a patchy to diffuse transepithelial labelling of the cells (Fig. 2). In the thicker non-keratinizing squamous epithelium replacing the typical OKC lining, the most consistent change was the loss of bcl2 staining, which occurred in 10/11 cases, if cases K5 and S15c are also considered (Table 2, Fig. 3e, f). These latter cases displayed areas of non-keratinizing squamous epithelium with and without basal palisading, and only the latter area demonstrated loss of bcl2 staining (Fig. 3). Altogether, four specimens of three patients showed partially or fully retained palisading of the basal layer of the thicker non-keratinizing epithelial lining (Cases K1 - biopsy and surgically removed cyst, K5 and S15c, Table 2, Fig. 3), and in three samples, these areas seemed to show the typical OKC epithelium staining pattern with bcl2, CK10 and CK17 positivity (Fig. 3). The loss of CK17 positivity was present in 4/11 cases and in the non-palisading part of two further OKCs, and was reduced in extent and intensity in further three cases. One case demonstrating only palisading non-keratinizing squamous epithelium and a further case with no palisading showed at least patchy transepithelial CK17 staining. Although CK10 was negative in 3 altered epithelia, and showed minor areas of negativity in the remaining, focal lack of staining was also seen in typical parakeratotic OKC lining, therefore this negativity could not be considered of much diagnostic help. Similarly, CK19 showed no consistent changes in the regenerative type altered epithelium.

## Discussion

OKCs whether parts of the Gorlin-Goltz syndrome or sporadic, are slowly growing cysts of the jaws leading to bone destruction, and requiring some type of treatment. Conservative treatment includes opening of the cyst, its drainage / marsupialization and removal after a reduction in size. Enucleation may be the first choice for smaller lesions and larger lesions may require more radical surgery. Because of these therapeutic consequences, their adequate recognition and diagnosis are essential.

Uncomplicated OKCs have a very characteristic morphology that distinguishes them from all other neoplastic, developmental or inflammatory cysts. However, inflammation or previous manipulation, such as a biopsy or marsupialization may lead to an alteration of the typical and diagnostic epithelium and may make the diagnosis difficult or impossible, depending on the size of the specimen. The thin, distinctively parakeratotic epithelial lining of OKCs may become a thick non-keratinizing squamous epithelium loosing also the basal palisading and waviness of the surface. In the present series, no OKC demonstrated complete loss of the original epithelial features, but some displayed sufficient areas of altered non-keratinizing epithelium to allow a sampling with exclusively this type of non-specific cyst lining, i.e. harbouring the possibility of missing or questioning the diagnosis of OKC.

The fact that inflammation leads to an alteration in the typical epithelial lining of OKCs and that this change may alter the IHC patterns of staining has been recognized earlier. In their cytokeratin profiling of OKCs, Aragaki and colleagues restricted the interpretation of staining to non-inflamed areas without reactive epithelial changes [14]. However, we are unaware of previous studies aiming to characterize the altered epithelium and compare it with the typical lining of OKCs.

The phenomenon that decompression therapy for about 12 months leads to the alteration of the OKC epithelium has been recognized earlier. (Some caution is needed with the results reported earlier, as OKC was used in a broader sense at the time of these reports, and cysts belonging to the now separate entity of OOC were also included; the numbers quoted here reflect only the subset that would correspond to OKC today.) Marker and colleagues have reported that 14 of their 18 OKCs have converted from typical OKC epithelium to non-keratinizing squamous epithelium [17] and similar results had been reported or summarized by others [1, 18]. Beside the morphological change of the epithelium after long term draining, a parallel loss of CK10 staining was also reported in 9/14 cases of OKC [19]. This loss of CK10 positivity was also suggested as a possible means of controlling the epithelial alteration in cytology samples during decompression, especially because CK10 positivity of OKC is manifested in the superficial layers [19, 20]. In contrast to these results, Tsuji et al. reported CK10 positivity in only about 10% of 25 OKCs studied, with a staining restricted to the superficial layer (as opposed to OOCs being generally positive) [15]. Aragaki and colleagues, in their elaborate cytokeratin profiling work of 20 OKCs and 20 OOCs, suggested that 7/20 OKCs were CK10 positive in the superficial layers of the epithelium lining the cysts [14]. These latter figures question the ability of CK10 staining and its loss to reflect epithelial changes in OKCs. In the present study, CK10 positivity was present in the superficial layers of all examples of typical OKC lining, but was segmental with varying proportions of unstained areas, giving ground to possible negativity in smaller biopsy specimens. The altered non-keratinized epithelium demonstrated lack of CK10 staining, but generally with areas of retained staining, therefore without a distinctive feature when compared with typical OKC epithelium.

According to published works and the present study results, basal bcl2, varying transepithelial CK17 and CK19 and often segmental superficial CK10 staining characterize OKCs. This is a distinctive pattern, that (with the exception of CK19 staining showing an overlap with other odontogenic cysts) can help in the differential diagnosis, as the most common odontogenic cysts (radicular and dentigerous ones) as well as the OOCs do not exhibit this pattern. The change in epithelium from typical thin parakeratotic to thicker non-keratinizing has already been reported to be associated with preoperative long-term drainage commonly leading to reduction in size. A similar focal change may be seen even a few months after biopsy or in association with inflammation.

Although our study is limited by the number of cases available, the flowing conclusions may be formulated. The typical basal/suprabasal staining with bcl2 generally vanishes from the altered non-keratinizing epithelium of OKCs observed either after biopsy with or without drainage or in association with inflammation, and this may at occasions interfere with the proper histological diagnosis. (As bcl2 positivity has been related to the alteration of the SHH pathway, its loss suggests that the altered epithelium not only looks different but may also have a different evolutionary history.) The transepithelial CK17 staining is also often lost or reduced in the altered epithelium adding to the diagnostic difficulty. On the other hand, CK10 and CK19 staining patterns did not demonstrate a diagnostically useful alteration, despite previous reports on CK10 loss. Although some loss with the latter antibody was present, the original OKC lining also demonstrated areas devoid of staining. As non-keratinizing areas with retained basal palisading seemed to display some of the typical OKC staining pattern, it may represent a transitional form in the epithelial change; the presence of such intermediate epithelium may therefore favour an OKC diagnosis even in the absence of the fully typical epithelium. On the other hand, the lack of a typical epithelial and IHC staining pattern alone is not sufficient to exclude the presence of OKC if the sampling is not generous enough.
